# Genetics of skin color variation in Europeans: genome-wide association studies with functional follow-up

**DOI:** 10.1007/s00439-015-1559-0

**Published:** 2015-05-12

**Authors:** Fan Liu, Mijke Visser, David L. Duffy, Pirro G. Hysi, Leonie C. Jacobs, Oscar Lao, Kaiyin Zhong, Susan Walsh, Lakshmi Chaitanya, Andreas Wollstein, Gu Zhu, Grant W. Montgomery, Anjali K. Henders, Massimo Mangino, Daniel Glass, Veronique Bataille, Richard A. Sturm, Fernando Rivadeneira, Albert Hofman, Wilfred F. J. van IJcken, André G. Uitterlinden, Robert-Jan T. S. Palstra, Timothy D. Spector, Nicholas G. Martin, Tamar E. C. Nijsten, Manfred Kayser

**Affiliations:** Department of Forensic Molecular Biology, Erasmus MC University Medical Center Rotterdam, Rotterdam, The Netherlands; Queensland Institute of Medical Research, Brisbane, Australia; Department of Twin Research and Genetic Epidemiology, King’s College London, London, UK; Department of Dermatology, Erasmus MC University Medical Center Rotterdam, Rotterdam, The Netherlands; Department of Biology, Indiana University-Purdue University Indianapolis, Indianapolis, IN 46202 USA; Section of Evolutionary Biology, Department of Biology II, University of Munich LMU, Planegg-Martinsried, Germany; Dermatology Research Centre, The University of Queensland, School of Medicine, Translational Research Institute, Brisbane, Queensland Australia; Department of Internal Medicine, Erasmus MC University Medical Center Rotterdam, Rotterdam, The Netherlands; Department of Epidemiology, Erasmus MC University Medical Center Rotterdam, Rotterdam, The Netherlands; Centre for Biomics, Erasmus MC University Medical Center Rotterdam, Rotterdam, The Netherlands; Department of Biochemistry, Erasmus MC University Medical Center Rotterdam, Rotterdam, The Netherlands; CNAG-Centro Nacional de Análisis Genómico, Parc Científic de Barcelona, 08028 Barcelona, Spain

## Abstract

In the International Visible Trait Genetics (VisiGen) Consortium, we investigated the genetics of human skin color by combining a series of genome-wide association studies (GWAS) in a total of 17,262 Europeans with functional follow-up of discovered loci. Our GWAS provide the first genome-wide significant evidence for chromosome 20q11.22 harboring the *ASIP* gene being explicitly associated with skin color in Europeans. In addition, genomic loci at 5p13.2 (*SLC45A2*), 6p25.3 (*IRF4*), 15q13.1 (*HERC2/OCA2*), and 16q24.3 (*MC1R*) were confirmed to be involved in skin coloration in Europeans. In follow-up gene expression and regulation studies of 22 genes in 20q11.22, we highlighted two novel genes *EIF2S2* and *GSS*, serving as competing functional candidates in this region and providing future research lines. A genetically inferred skin color score obtained from the 9 top-associated SNPs from 9 genes in 940 worldwide samples (HGDP-CEPH) showed a clear gradual pattern in Western Eurasians similar to the distribution of physical skin color, suggesting the used 9 SNPs as suitable markers for DNA prediction of skin color in Europeans and neighboring populations, relevant in future forensic and anthropological investigations.

**Electronic supplementary material:**

The online version of this article (doi:10.1007/s00439-015-1559-0) contains supplementary material, which is available to authorized users.

## Introduction

While the principal genes influencing eye and hair color are now largely identified, current knowledge on the genetic basis of skin color variation is still limited (Liu et al. 2013). A better understanding of human skin color genetics is highly relevant for medicine, i.e., due to the relationship with many skin diseases such as skin cancer (Chen et al. 2014); evolutionary biology, i.e., due to the widely assumed environmental adaptation in skin color via positive selection (Sturm 2009); as well as anthropological and forensic applications of DNA predicting skin color of unknown individuals, including deceased modern and archaic humans, and unknown perpetrators to provide investigative leads (Kayser 2015; Kayser and de Knijff 2011). Recently, we reported a comprehensive candidate gene study identifying two genes (*BNC2* and *UGT1A*) influencing skin color variation in Europeans (Jacobs et al. 2012). To search for additional DNA variants involved in European skin color variation, the International Visible Trait Genetics (VisiGen) Consortium conducted a series of genome-wide association studies (GWASs) followed by a replication analysis in a total of 17,262 Europeans (Table S1) from three discovery cohorts including: the Rotterdam Study (RS) *n* = 5857 from the Netherlands, the Brisbane Twin Nevus Study (BTNS) *n* = 3459 of European descent from Australia, and the TwinsUK study, *n* = 2668 from the United Kingdom. Further replication was conducted in the National Child Development Study (NCDS), *n* = 5278 from the United Kingdom. Skin color phenotypes included quantitative skin color saturation (S), perceived skin darkness (PSD), the Fitzpatrick scale (FPS) of skin sensitivity to sun (Fitzpatrick 1988), and self-reported skin color darkness (note that different phenotypes were available in different cohorts, for details see Table S1). Functional follow-up was performed on the genomic regions identified by the skin color GWAS.

## Results and discussion

A total of five distinct genomic regions were identified that harbored DNA variants associated with skin color at the genome-wide significant level (*p* value <5 × 10^−8^) including: 5p13.2 containing *SLC45A2*; 6p25.3 containing *IRF4*; 15q13.1 containing *OCA2* and *HERC2*; 16q24.3 containing *MC1R*; and 20q11.22 spanning ~1.5 Mb containing *ASIP* (Table S2; Figure S1). Association signals observed at all five loci were highly significantly replicated in NCDS (*p* value <1 × 10^−6^, Table S3).

In a multivariate analysis of the 9 top-associated single-nucleotide polymorphisms (SNPs) from 9 different genes (Table 1), all except *SLC24A5* rs2924567 showed significant and independent effects on both saturation and PSD in RS, which is largely in line with previous findings for eye color (Eiberg et al. 2008; Liu et al. 2009, 2010; Sturm et al. 2008) and hair color (Branicki et al. 2011; Han et al. 2008). The 9 SNPs together explained a substantial proportion of the phenotypic skin color variance depending on phenotype and population (from 3.3 % saturation in RS to 16.3 % for PSD in BTNS), which is much larger than typical findings from GWAS of human complex traits such as body height [e.g., 9500 SNPs together explain up to 29 % phenotypic variance (Wood et al. 2014)] but much smaller than that of human eye color [12 SNPs from 10 genomic loci explain ~50 % phenotypic variance (Liu et al. 2010)]. *HERC2* rs12913832 displayed the strongest effect on skin color, which was larger on PSD (*R*^2^ = 5.02 % in RS) than on saturation (*R*^2^ = 0.497 % in RS). SNP allele frequencies between RS and BTNS were largely similar, with the exception of *IRF4* rs12203592, for which the lighter color-associated T allele had a 2.5-fold higher frequency in BTNS (0.234) compared to in RS (0.092). Consequently, *IRF4* rs12203592 was found as the second influential genetic factor (*R*^2^ = 3.63 %) for PSD in BTNS after *HERC2* rs12913832 (*R*^2^ = 5.38 %). *SLC45A2* rs183671 was in strong linkage disequilibrium (LD *r*^2^ = 0.90) with a known coding variant rs16891982 (F374L) from the same gene (Duffy et al. 2010) and both SNPs showed very similar effects in all cohorts (Table S2). *BNC2* rs10756819 was not genome-wide significant but showed a consistent and nominally significant effect in all studied cohorts (*p* value <0.05); this therefore replicated our previous finding from a candidate gene study (Jacobs et al. 2012) suggesting that *BNC2* influences subtle variation in skin color. A detailed analysis for *MC1R* SNPs was conducted in RS (Table S4), where several compound *MC1R* markers (MC1R-R, MC1R-r, and MC1R-All) were tested for association with saturation and PSD. Consistent with previous findings on red hair color (Liu et al. 2011), the MC1R-R consisting of 3 high-penetrance variants (rs1805007 (R151C), rs1805008 (R160W), rs1805009 (D294H)) showed the most significant association with skin color phenotypes (*p* value = 2.92 × 10^−16^ for skin saturation and *p* value = 2.23 × 10^−13^ for PSD). In terms of the explained phenotypic variance, replacing the top-associated chr16 SNP rs4268748 by MC1R-R increased the explained *R*^2^ marginally (0.1 % for saturation and 0.3 % for PSD). A pair-wise SNP–SNP interaction analysis using all 9 variants (rs4268748 was replaced by MC1R-R) in RS did not reveal any significant interaction effects (Table S5).

**Table 1:** Genetic factors explaining skin color variation in three European studies RS, BTNS, and TwinsUK

SNP_EA	Chr_gene	Skin saturation (RS)				PSD (RS)			PSD (BTNS)				Fitzpatrick (TwinsUK)		
		*f*EA	Beta	*p*	*R* ^2^ %	Beta	*p*	*R* ^2^ %	*f*EA	Beta	*p*	*R* ^2^ %	*f*EA	Beta	*p*
Age (years)			−0.002	2.95E−262	17.537	−0.013	3.21E−106	8.916							
Sex (female)			0.041	8.22E−185	10.687	0.199	3.35E−59	3.711		−0.032	0.142	<0.01			
rs183671_T	5_SLC45A2	0.021	0.021	4.46E−10	0.439	0.178	1.51E−09	0.482	0.029	0.505	1.64E−14	2.998	0.056	0.282	9.08E−05
rs12203592_T	6_IRF4	0.092	−0.011	2.26E−10	0.478	−0.055	1.64E−04	0.201	0.234	−0.220	1.09E−17	3.627	0.145	−0.157	1.12E−03
rs10756819_G	9_BNC2	0.332	0.005	4.40E−06	0.261	0.027	2.50E−03	0.130	0.325	0.051	3.03E−02	0.243	0.330	0.071	6.13E−03
rs1393350_A	11_TYR	0.235	−0.004	2.37E−04	0.163	−0.046	4.24E−06	0.304	0.292	−0.081	2.24E−03	0.573	0.282	−0.136	3.54E−07
rs17128291_G	14_SLC24A4	0.157	−0.003	4.24E−02	0.048	−0.038	9.55E−04	0.147	0.152	−0.067	2.95E−02	0.241	0.151	−0.044	2.05E−01
rs12913832_A	15_HERC2	0.200	0.008	1.08E−10	0.497	0.209	9.02E−78	5.029	0.217	0.275	1.59E−24	5.376	0.280	0.075	2.10E−02
rs2924567_T	15_SLC24A5	0.374	−0.002	2.11E−02	0.060	−0.016	7.34E−02	0.044	0.368	−0.017	4.78E−01	0.029	0.349	0.005	8.63E−01
rs4268748_C	16_MC1R	0.277	−0.009	2.59E−15	0.758	−0.049	2.15E−07	0.376	0.294	−0.158	2.84E−10	2.161	0.315	−0.214	3.62E−16
rs6059655_A	20_RALY/ASIP	0.079	−0.013	6.36E−13	0.608	−0.072	4.22E−06	0.278	0.099	−0.169	8.58E−06	1.057	0.096	−0.220	1.27E−07
Phenotypic variance (*R* ^2^ %) explained by SNPs					3.313			6.992				16.305			
*R* ^2^ % explained by all factors investigated					31.536			19.620				16.305			

Saturation: quantitative measure derived from high-resolution digital photographs; PSD: three-level perceived skin darkness

For genome-wide significant hits, only the most significant SNP per gene locus (*SLC45A2*, *IRF4*, *HERC2*, *MC1R*, and *ASIP*) is included

For non-genome-wide significant hits, 4 SNPs in 4 known skin color genes are also included (*BNC2*, *TYR*, *SLC24A5*, and *SLC24A4*)

In BTNS, age varies in a small range (12–14 years)

Multivariate analysis in RS and BTNS and univariate analysis in TwinsUK

*EA* effect allele, *fEA* frequency of the effect allele, *R* ^2^ *%* percentage of phenotypic variance explained from multivariate analysis

All 9 SNPs listed in Table 1 were used to construct a genetically inferred skin color score in 940 samples from 54 worldwide populations (HGDP-CEPH samples), which showed a spatial distribution with a clear gradual increase in skin darkness from Northern Europe to Southern Europe to Northern Africa, the Middle East and Western Asia (Figure S2); in agreement with the known distribution of skin color across these geographic regions. Outside of these geographic regions, the inferred skin color score appeared rather similar (i.e., failing to discriminate), despite the known phenotypic skin color difference between generally lighter Asians/Native Americans and darker Africans. This demonstrates that although these 9 SNPs can explain skin color variation among Europeans, they cannot explain existing skin color differences between Asians/Native Americans and Africans. Therefore, these differences in skin color variation may partly be due to different DNA variants not identifiable by this European study with restricted genetic origin.

**Fig. 1:**
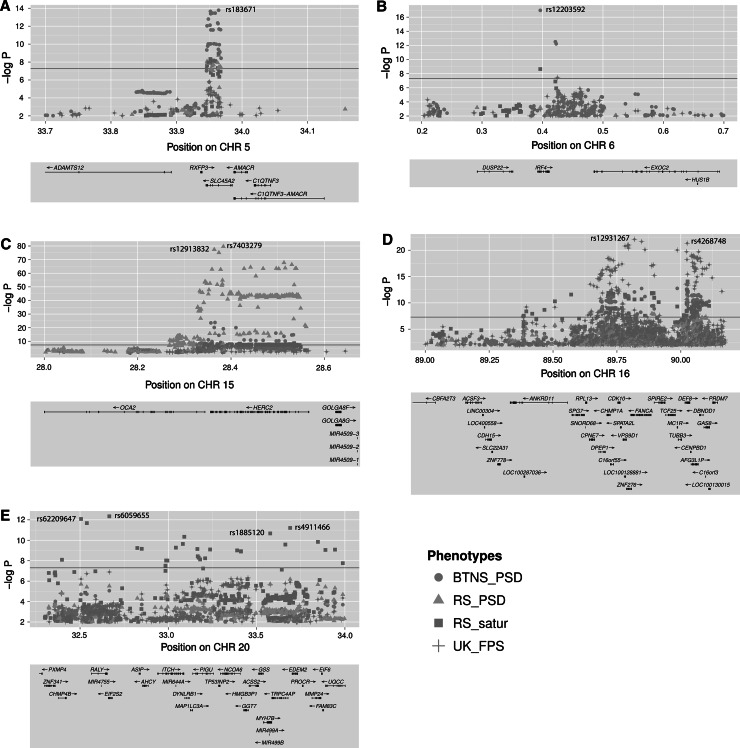
Regional Manhattan plots for skin color phenotypes in the Rotterdam Study, the Brisbane Twin Nevus Study, and the TwinsUK study. **a** chromosome 5p13.2 (33.7–34.2 Mb) containing *SLC45A2*; **b** chromosome 6p25.3 (0.2–0.7 Mb) containing *IRF4*; **c** chromosome 15q13.1 (28.0–28.7 Mb) containing *OCA2* and *HERC2*; **d** chromosome 16q24.3 (89.0–90.2 Mb) containing *MC1R*; and **e** a large region on chromosome 20q11.22 spanning ~1.5 Mb (32.3–34.0 Mb) containing *ASIP*. The −log10 *p* values of all SNPs are plotted against their physical positions (hg19). The *blue horizontal line* stands for the *p* value threshold of 5 × 10^−8^. *p* value dots are represented in *colors* and *shapes* indicating different phenotypes from different study cohorts (*plink circle* perceived skin darkness in BTNS, *green triangles* perceived skin darkness in RS, *blue squares* quantitative skin color saturation in RS, and *purple pluses* Fitzpatrick scales in TwinsUK). The physical positions of all known genes in the regions are aligned (color figure online)

For four of the five highlighted genomic regions, i.e., 5p13.2 (Fig. 1a), 6p25.3 (Fig. 1b), 15q13.1 (Fig. 1c), and 16q24.3 (Fig. 1d), the genes responsible for the noted skin color association signals are well documented for their involvement in human pigmentation traits, i.e., *SLC45A2*, *IRF4*, *OCA2*/*HERC2*, and *MC1R*, respectively (Liu et al. 2013). However, from previous studies it is much less clear which gene(s) in the 20q11.22 region may functionally explain the observed SNP association with skin color (Liu et al. 2013). Unlike the other four regions, the top-associated SNP (rs6059655) in 20q11.22 (Fig. 1e) was genome-wide significant only for quantitative skin color saturation (*p* = 6.36 × 10^−13^ in RS), and was less significant for PSD (*p* value = 4.22 × 10^−6^ in RS; *p* value = 8.58 × 10^−6^ in BTNS) and FPS (*p* value = 1.27 × 10^−7^ in TwinsUK, Table S2). Among all other 20q11 SNPs with *p* values smaller than 1 × 10^−6^, rs1885120 within *MYH7B* and rs910873 within *PIGU* have been previously associated with melanoma risk (Brown et al. 2008), and an intergenic SNP rs4911466 has been associated with sun burning, freckling, red hair, and skin sensitivity to sun (Sulem et al. 2008). The association signals noted at 20q11.22 span a large haplotype block of ~1.5 Mb containing 22 known genes, among which *ASIP*, a gene encoding the agouti signaling protein, is assumed to be involved in melanogenesis (Suzuki et al. 1997). However, variants from coding regions of *ASIP* may not explain the observed association as previously suggested (Sulem et al. 2008). The SNP rs6059655 in intron 8 of *RALY* is ~182 kBp (hg19) upstream of *ASIP*. All other SNPs in this region showing association signals (*p* value <1 × 10^−6^) with skin color phenotypes were in moderate or high linkage disequilibrium with rs6059655 (LD *r*^2^ > 0.4 in our European data). However, none of them displayed any significant independent association at the genome-wide level after conditioning for the rs6059655 genotype (all *p* values >0.001). Haplotype and SNP interaction analyses at 20q11 did not reveal more significant association signals for other SNPs than rs6059655 alone (Figure S3).

Aiming to functionally explain the skin color association signal observed in the 20q11.22 region, we investigated the expression patterns of 22 genes in this region. First, we analyzed whole-transcriptome sequencing data we obtained from 6 skin melanocytic cell lines (MCLs) of two light, one moderate, and three dark-pigmented individuals, followed by confirmatory analysis via reverse transcriptase quantitative PCR (RT-qPCR) of the 22 genes in the 6 MCLs as well as in 29 skin epidermal samples (SESs) derived from 12 dark and 17 light skin colored donors [detailed sample information is provided elsewhere (Visser et al. 2014)] (Table 2). In the whole-transcriptome sequencing data from the MCLs, we observed higher expression levels in the dark- and moderate-pigmented melanocyte MCLs than in the light-pigmented ones for 8 of 22 genes (*RALY*, *EIF2S2*, *AHCY*, *ITCH*, *MAP1LC3A*, *EDEM2*, *EIF6* and *UQCC*). Expression of 3 genes (*ASIP*, *MYH7B* and *FAM83C*) was not detected, while for the remaining 11 genes the expression levels were not observed to be statistically significantly different between the dark- and moderate-pigmented MCLs and the light-pigmented MCLs. The observed differential gene expression was confirmed by RT-qPCR for 5 of the 8 genes (*RALY*, *EIF2S2*, *AHCY*, *ITCH*, and *EDEM2*) in the 6 MCLs as well as in the 29 SESs. In addition, the genes *NCOA6* and *GSS* that were initially not highlighted by the RNA sequencing results, showed differential expression between light- and dark-pigmented samples in the MCLs as well as in the SESs for *NCOA6*, and in the SESs only for *GSS* ( Table 2, Ct values and *p* values are indicated in Table S6 and Table S7). Expression of 4 of the 22 genes (*ASIP*, *MYH7B*, *MMP24* and *FAM83C*) was not detected in the MCLs or in the SESs (Table 2, Ct values and *p* values are indicated in Table S6 and Table S7).

**Table 2:** Expression profile of 22 genes at 20q11.22 in 6 melanocyte cell lines and 29 skin epidermal samples from individuals of different pigmentation status

	Melanocyte cell lines		Skin epidermal samples	rs1885120 in melanocyte cell lines	Combined *p* value
	RNA-seq	qPCR	qPCR	qPCR	
RALY	+	−	++	−	0.157
EIF2S2	++	+	++	−	0.017
ASIP	nd	nd	nd	nd	Nd
AHCY	+	−	+	−	0.312
ITCH	+	++	++	−	0.047
DYNLRB1	−	−	−	−	1.0
MAP1LC3A	+	−	−	−	0.871
PIGU	−	−	−	−	0.642
TP53INP2	−	−	−	−	0.759
NCOA6	−	++	+	−	0.083
GGT7	−	−	−	−	0.811
ACSS2	−	−	−	−	0.749
GSS	−	−	++	++	0.0007
MYH7B	nd	nd	nd	nd	Nd
TRPC4AP	−	−	−	−	0.426
EDEM2	+	+	+	−	0.211
PROCR	−	−	+	−	0.327
MMP24	−	nd	nd	nd	Nd
MMP24-AS1	−	−	−	−	1.0
EIF6	++	−	−	+	0.045
FAM83C	nd	nd	nd	nd	Nd
UQCC	++	−	−	+	0.039

Transcription of 22 genes at 20q11.22 was measured in 6 melanocyte cell lines, two light-pigmented (LP22, LP89), one medium-pigmented (MP01), and three dark-pigmented (DP74, DP80 and DP83) ones using whole-transcriptome sequencing (1st data column) and results were tested for confirmation with RT-qPCR (2nd data column). Transcription of the 22 genes in 29 skin epidermal samples from 17 light-skinned and 12 dark-skinned volunteers was measured using RT-qPCR (3rd data column). Correlation between rs1881520 and the expression of the 22 genes at 20q11.22 in the 6 melanocyte cell lines (4th data column). *p* values of the 4 independent transcription analyses are combined to determine their significance, corrected by the number of genes tested (5th column). Correlations are denoted according to statistical significance: ^−^ (*p* > 0.05), ^+^ (*p* < 0.05), ^++^ (*p* < 0.01), and *nd* not detected

As non-coding SNPs have been reported to be involved in transcriptional regulation of nearby pigmentation genes (Guenther et al. 2014; Praetorius et al. 2013; Visser et al. 2012, 2014, 2015), we next investigated the correlation between the genotypes of rs6059655 and the transcription of the 22 genes at 20q11.22 in the 6 MCLs and in the 29 SESs. However, both sample sets did not have enough genotypic variation for rs6059655 or its LD partners, therefore, no genotype-expression correlation was observed for any of the 22 genes tested with one exception. In the 6 MCLs, the genotypes of the LD SNP rs1885120 (LD *r*^2^ = 0.82) were statistically significantly correlated with the expression of *GSS* (*p* value <0.01), and potentially significantly with the expression of *EIF6* and *UQCC* (*p* value <0.05) (Table 2, Ct values and *p* values are indicated in Table S8).

As we found rs1885120 and potentially rs6059655 correlating with the expression of one or more genes at 20q11.22, we further checked if the physical positions of any noted SNPs might coincide with a regulatory element that affects expression levels of the correlated regional gene(s). Using a combination of two newly obtained and several previously published ChIP-seq data sets (Li et al. 2012; Rosenbloom et al. 2013; Strub et al. 2011), we profiled the chromatin at 20q11.22 for features of regulatory elements (Figure S4). This analysis identified many different promoter and (potential) regulatory elements within 20q11.22, indicating that the region is indeed transcriptionally active in both epidermal melanocytes and epidermal keratinocytes. However, none of the regions identified with regulatory potential coincided with the physical positions of any noted SNPs.

**Fig. 2:**
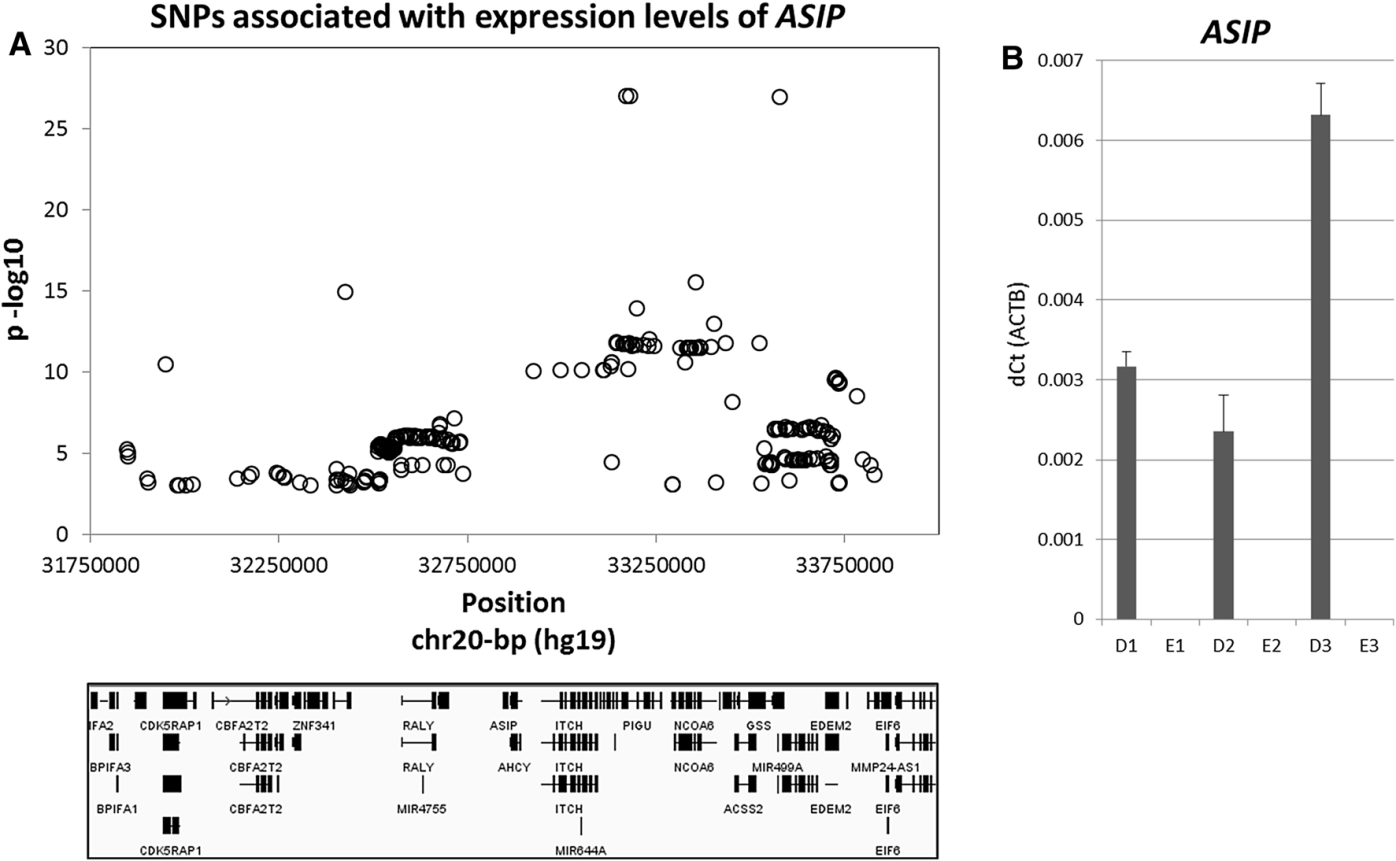
Expression of *ASIP* in full, dermal, and epidermal layers of skin. **a** Plot of eQTL analysis on *ASIP*, where expression of *ASIP* is strongly associated with pigmentation variants rs17305573, rs910873 and rs1885120 in skin full-layer biopsy samples. **b** *ASIP* is exclusively expressed in the dermal layer of skin, and not in the epidermal layer of skin (*nd* not detected). Samples derived from the dermal layer are denoted with ‘*D*’, samples derived from the epidermal layer are denoted with ‘*E*’. Sample 1 has the rs1885120-CC genotype, with a dark skin phenotype, sample 2 has the rs1885120-CC genotype, with a light skin phenotype, sample 3 has the rs1885120-CT genotype, with a light skin phenotype

To further investigate the expression patterns of the 22 genes at 20q11.22 and their (potential) correlation with pigmentation-SNP genotypes, we checked the expression quantitative trait locus (eQTL) data in the publically available Multiple Tissue Human Expression Resource (MuTHER) Study database (Nica et al. 2011). We found for skin biopsy samples a highly significant association between the expression of *ASIP* (HumanHT-12 array probe ILMN_1791647 targeting exon 3 of *ASIP*) and the SNPs rs1885120, rs910873 and rs17305573 (*p* value = 1 × 10^−26^) (Fig. 2a; Table S9), while for the expression of 8 genes at 20q11.22 (*EIF2S2*, *ITCH*, *MAP1LC3A*, *GGT7*, *EDEM2*, *PROCR*, *EIF6*, and *FAM83C* of which *EIF2S2*, *ITCH*, *MAP1LC3A*, *EDEM2*, *PROCR* and *EIF6* were highlighted by at least one of our previous expression analyses) more modest associations with SNP genotypes were found. For these 8 genes, the expression-associated SNPs resulting from the eQTL analysis do, however, not overlap with our GWAS results (Table S9). Notably, in the MuTHER project, full-layer skin samples were used while in our study only epidermal samples were applied. The difference between our data and that obtained with the MuTHER-eQTL analysis might therefore be explained by expression of *ASIP* in the skin dermis rather than in the melanocytes or keratinocytes located in the skin epidermis.

To test this hypothesis, we analyzed expression patterns of *ASIP* in the dermis and the epidermis of skin biopsy samples obtained from three individuals. This analysis indeed revealed a robust expression of *ASIP* in the dermal samples (not containing melanocytes), whereas in the melanocyte-containing epidermal samples *ASIP* was not detected (Fig. 2b). In addition, expression of *ASIP* was found to be higher in the dermal sample from the rs1885120-CT heterozygote carrier than in the two samples from the rs1885120-CC homozygote carriers, which confirms the findings of the MuTHER eQTL study. It is known that not only epidermal but also dermal components contribute to normal skin pigmentation. For example, dermal fibroblasts are involved in the secretion of several (paracrine) factors that modulate signaling pathways that are involved in melanocyte function and consequent skin pigmentation (Yamaguchi and Hearing 2009). As *ASIP* is a well-known antagonist of the receptor molecule MC1R located on the cell surface of melanocytes (Suzuki et al. 1997), it is possible that *ASIP* becomes secreted by dermal components like fibroblasts to interact with MC1R. Furthermore, it has been suggested that the epidermal melanin unit not only consists of melanocytes and keratinocytes, but also involves Langerhans cells present in the epidermis that also exist in the papillary dermis (Jimbow et al. 1991; Nordlund 2007). Several human pigmentation disorders such as ceruloderma, a type of dermal melanosis, and forms of post-inflammatory hyperpigmentation are due to defects in the dermal layer, indicating that human pigmentation is indeed not exclusively regulated in the epidermis, but also in the dermis (Ortonne 2012). Additionally, a melanocyte reservoir for hair and skin (re-)pigmentation consisting of melanocyte stem cells (MelSCs) is located in specific compartments of hair follicles in the dermis (Nishimura 2011). Therefore, it might also possible that *ASIP* is expressed in these MelSCs, and becomes silenced upon differentiation of the melanocytes in the epidermis.

*P* values from all above experiments were then combined using Fisher’s method, resulting into a list of 6 genes at 20q11.22, namely *ASIP*, *EIF2S2*, *ITCH*, *GSS*, *EIF6* and *UQCC* that are indicated with significant functional evidence to be involved in human skin color variation (combined *p* value <0.05, Table 2). Although the location of rs6059655, as well as previous studies on the agouti-yellow (A^y^) deletion in mice (Michaud et al. 1994), suggest an involvement of the *RALY* gene in skin pigmentation, our data did not provide enough evidence supporting *RALY* as a functional human skin color gene (combined *p* value = 0.15). However, its upstream-neighboring gene *EIF2S2*, of which the expression was significantly (combined *p* value = 0.02) correlated with pigmentation phenotypes, was also deleted in the same A^y^ mutation as *RALY*, and was shown to be involved in other A^y^ mutation phenotypes (Heaney et al. 2009). Moreover, the gene with the highest significance was *GSS* (combined *p* value <0.001, Table 2), which encodes the glutathione synthetase involved in the catalyzation of the second step of the glutathione (GSH) biosynthesis. GSH is a highly important cellular antioxidant with multiple cellular functions and major effects on melanogenesis within melanocytes. For example, GSH was shown to play a crucial role in the switching between eumelanogenesis and pheomelanogenesis by interacting with the tyrosinase enzyme (del Marmol et al. 1993) and by reacting with dopaquinone in the tyrosinase pathway (Ito 2003; Jara et al. 1988). Moreover, GSH was shown to be involved in the oxidative processes of melanin formation (Panzella et al. 2014), and was differentially detected in skin biopsy samples of different skin color (Halprin and Ohkawara 1966). Recently, an experimental study revealed that oral administration of GSH induces depigmentation of skin (Arjinpathana and Asawanonda 2012). These lines of evidence, together with our association and expression data, support an important role of *GSS* in skin coloration. Based on our analysis of the chromatin profile at the region around rs6059655, it seems unlikely that this SNP acts as an enhancer element that regulates transcription of *GSS* (or another pigmentation gene). Instead, other (yet unknown) markers in LD might do so [as was shown for *BNC2* (Visser et al. 2014)]. Alternatively, rs6059655 might be involved in DNA folding or it could tag an indel.

In summary, our replicated GWAS of quantitative skin color provides the first genome-wide significant evidence for one or more common DNA variants at 20q11.22 being explicitly associated with skin coloration in Europeans. Furthermore, this study highlights additional variants associated at the genome-wide significant level with skin color arising from four additional regions containing genes known to be involved in the determination of pigmentation (5p13.2 containing *SLC45A2*, 6p25.3 containing *IRF4*, 15q13.1 containing *OCA2* and *HERC2*, and16q24.3 containing *MC1R*). A combination of 9 SNPs from 9 pigmentation genes was found useful to DNA-predict skin color in Europeans and neighboring populations. Functional analyses prioritized two genes at 20q11.22, *EIF2S2* and *GSS*, as the most likely novel candidates responsible for the genetic association we observed in this region; both genes were significantly differentially expressed in the skin epidermis. We further showed that *ASIP* is not expressed in the epidermis containing the pigment layer, but instead in the skin dermis. Consistent with the known biology of melanocyte cell regulation (Yamaguchi and Hearing 2009), our data suggest that skin color is regulated not only in the pigment layer of the epidermis, but also from within the dermal layer of skin. Seen together, these findings represent a step forward in the understanding of the genetic basis of pigmentation variation in humans and are relevant to DNA phenotyping of skin color for forensic and anthropological applications.

## Materials and methods

### Rotterdam study (RS)

The Dutch European RS (Hofman et al. 2013) is a population-based prospective study consisting of a main cohort and two extensions. The RS is ongoing since 1990 and currently includes 14,926 participants living in a particular suburb of Rotterdam in the Netherlands. The Medical Ethics Committee of the Erasmus University Medical Center approved the study protocol and all participants provided written informed consent. The current study includes 5857 participants of Northwestern European ancestry, with microarray genotype data and digital photographs available. No exclusions have been made on skin-related diseases. We used the same set of photographs as obtained and described in our previous eye color GWAS (Liu et al. 2010). Skin color phenotypes were derived and described in detail in a previous study (Jacobs et al. 2012); here, we focus on skin saturation (mean 0.524, SD 0.063, min 0.278, max 0.808) derived from digital photos and three-level perceived skin darkness graded by a dermatologist (very white 14.84 %, white 73.68 %, white-to-olive 11.48 %). Microarray genotyping was conducted using the Infinium II HumanHap550 K and Human 610 Quad Arrays of Illumina. Details on genotyping and quality controls are described elsewhere (Liu et al. 2010). Genotypes were imputed using the 1000-Genomes Project as the reference panel (Phase 1, integrated variant set across 1092 individuals, v2, March 2012) using the MaCH and minimac software packages. After all quality controls (MAF >0.01, marker call rate >0.97, and HWE >1 × 10^−6^), the final data set included 11,155,022 SNPs with imputation *R*^2^ >0.4.

### Brisbane Twin Nevus Study (BTNS)

The Australian BTNS has recruited adolescent twins, their siblings and parents over the past 22 years into an ongoing study of genetic and environmental factors contributing to the development of pigmented nevi and other risk factors for skin cancer. The proband twins are recruited at age 12 years via schools around Brisbane, Australia, and followed up at age fourteen. The sample is of Northern European origin (mainly Anglo-Celtic >95 %). All cases and controls gave informed consent to participation in this study, and the study protocol was approved by appropriate institutional review boards. Skin color at age 12–14 years was reported by participants as one of three categories: fair/light, medium, or olive/dark.

DNA samples from the BTNS were genotyped by the Scientific Services Division at deCODE Genetics, Iceland using the Illumina 610-Quad BeadChip; genotypes were called with the Illumina BeadStudio software. For the GWAS, we first applied filters to SNP data before evaluating genotyping quality per individual and excluded SNPs with a mean BeadStudio GenCall score <0.7. Next, we excluded poorly performing samples (call rate <0.95) and SNPs with call rate <0.95, Hardy–Weinberg equilibrium *p* < 10^−6^, or minor allele frequency <0.01. Following these exclusions, we compared self-reported with genotype-inferred family relationships, the latter based on genome-wide IBS sharing. Forty-eight families with pedigree errors were identified; 21 samples from these families were excluded to correct errors which could not be resolved. No SNPs or individuals showed segregation patterns inconsistent with Mendelian inheritance in >5 % of families and SNPs, respectively. Lastly, we excluded 88 individuals identified as outliers from populations of European descent through the estimation of genetic ancestry using EIGENSTRAT and data from eleven populations of the HapMap 3 and five Northern European populations genotyped by the GenomeEUtwin consortium. Following these exclusions, there remained 529,721 SNPs and 4296 individuals with genotype data for analysis. Imputation was undertaken with the use of the phased data from the HapMap samples of European ancestry (CEU; build 36, release 22) and MACH. After imputation quality controls (MACH *R*^2^ >0.4), this dataset included 2,558,980 SNPs.

### TwinsUK

The TwinsUK study included 2668 phenotyped participants (97 % female and all of Caucasian ancestry) within the TwinsUK adult twin registry based at St. Thomas’ Hospital in London. Twins largely volunteered unaware of the skin research interests at the time of enrolment and gave fully informed consent under a protocol reviewed by the St. Thomas’ Hospital Local Research Ethics Committee. Genotyping of the TwinsUK cohort was done with a combination of Illumina HumanHap300 and HumanHap610Q chips. Intensity data for each of the arrays were pooled separately and genotypes were called with the Illuminus32 calling algorithm, thresholding on a maximum posterior probability of 0.95 as previously described (Small et al. 2011). Imputation was performed using the IMPUTE 2.0 software package (https://mathgen.stats.ox.ac.uk/impute/impute.html) using haplotype information from the 1000 Genomes Project (Phase 1, integrated variant set across 1092 individuals, v2, March 2012). Imputed genotypes were subsequently converted into a MACH format (http://www.sph.umich.edu/csg/abecasis/MACH/tour/input_files.html) and analyzed with mach2qtl (http://www.sph.umich.edu/csg/abecasis/MACH/download/mach2qtl.source.V112.tgz).

### National Child Development Study (NCDS)

The NCDS is a cohort study of 17,000 people born in England, Scotland and Wales in a single week of 1958. The participants have been extensively phenotyped on multiple occasions, including a biomedical survey, which was designed to obtain objective measures of ill-health and biomedical risk factors to address a wide range of specific hypotheses relating to anthropometry: cardiovascular, respiratory and allergic diseases; visual and hearing impairment; and mental ill-health. In 2003, as part of the biomedical survey, 9377 participants completed an item on skin color, reporting it on a scale of “light”, “medium” or “dark”. Individuals were genotyped on both the Immunochip and Metabochip disease-centered SNP arrays. SNP sets were combined, data from duplicated SNP sets were merged, and monomorphic SNPs, SNPs exhibiting Hardy–Weinberg disequilibrium (*p* < 1 × 10^−6^) or SNPs with genotyping failure rate <0.98 were removed. A total of 298,548 SNPs were then available. Imputation for the regions of interest was performed using IMPUTE2 and the 1000 Genomes Phase1 phased dataset v3 dated 2010-11-23, and the reference set haplotypes estimated using SHAPEIT2 (ALL.integrated_phase1_SHAPEIT_16-06-14). The current study included 5278 NCDS participants for whom both skin color and genotype data were available.

### Statistical genetic analyses

GWAS were conducted in RS using linear regression assuming additive genetic effect and adjusted for sex, age, and 4 main dimensions from MDS analysis, where *p* values equal or smaller than 5 × 10^−8^ were considered to be genome-wide significant. Inflation factors were estimated as 1.015 for skin saturation and 1.011 for PSD and were adjusted using the genomic control method. The GWAS in TwinsUK cohort was conducted using mach2qtl v1.12 (http://www.sph.umich.edu/csg/abecasis/MACH/download/mach2qtl.source.V112.tgz). The genomic inflation factor was 1.01 for the Fitzpatrick scale in TwinsUK. Since the BTNS is comprised of twin families, a mixed model GWAS analysis was performed using MERLIN (Abecasis et al. 2002; Chen and Abecasis 2007), where PSD was treated as an interval trait. Inflation factor was estimated as 0.967 for PSD in BTNS.

Genome-wide Manhattan and Q–Q plots were generated using CRAN R library qqman v0.1.2. Regional Manhattan plots were constructed using software package locuszoom (Pruim et al. 2010). To access the overall genetic contribution on skin coloration, we conducted a multivariate analysis including 9 DNA variants from 9 genes, i.e., 5 highlighted in the present study including *RALY* rs6059655, *HERC2* rs12913832, *IRF4* rs12203592, *SLC45A2* rs183671, *MC1R* rs4268748 and 4 suggested in previous studies (Han et al. 2008; Jacobs et al. 2012; Lamason et al. 2005; Sulem et al. 2008) including *BNC2* rs10756819, *TYR* rs1393350, *SLC24A4* rs17128291, and *SLC24A5* rs2924567 (Table 1). Since both quantitative skin color saturation and the three-level PSD phenotypes were available in RS, the genetic effects on these two phenotypes could be compared. The multivariate analysis including sex, age, and 9 SNPs from 9 genes were conducted in RS and BTNS in an iterative manner to access the *R*^2^ change due to individual factors using R scripting, i.e., by adding one *x*-variable at a time to a linear model (lm function in R) in ascending order according to the *p* values from the multivariate analysis including all *x*-variables; the *R*^2^ estimated for the added *x*-variable is then the *R*^2^ difference between the current model and a previous model. We further inferred a skin color score for 940 samples from 54 populations in the HGDP database (http://www.cephb.fr/en/hgdp/diversity.php) using the sum of the number of darker skin-associated alleles weighted by the regression betas for skin saturation from the multivariate analysis.

Since *MC1R* polymorphisms are known to interact with each other through compound heterozygosity. A collapsed genotype analysis of 6 MC1R SNPs was conducted in RS (due to SNP availability). The MC1R-R genotype (wt/wt, wt/R, R/R) was collapsed from 3 highly penetrant causal variants, i.e., rs1805007 (R151C), rs1805008 (R160 W), and rs1805009 (D294H), based on a haplotype analysis. Other high-penetrance *MC1R* variants were too rare and did not pass imputation quality control: Y152OCH, N29insA, rs1805006 (D84E), and rs11547464 (R142H). The MC1R-r genotype (wt/wt, wt/r, r/r) was collapsed from 3 low penetrant variants rs1805005 (V60L), rs2228479 (V92 M), and rs1110400 (I155T). Another low penetrance variant (rs885479 (R163Q) was not available. The MC1R variants (including MC1R-R and MC1R-r) were separately tested for association with skin saturation and PSD in RS using linear regression adjusted for sex and age (Table S4). Conditional analyses were conducted for all associated regions conditioning on the genotype status of the top-associated SNP. Haplotype analyses were conducted using CRAN R library haplo.stats. SNP interaction analyses were conducted between SNPs listed in Table 1 and between all SNPs in the *MC1R* region and the *ASIP* region using a previously described F statistic (Liu et al. 2010). Gene transcription levels were compared between genotype carriers (wild-type vs. others) using a *t* test. A combined *p* value was derived for each gene by combining *p* values from *k* independent experiments using Fisher’s combined probability test, i.e., $X_{2k}^2\ - 2\sum\nolimits_{i = 1}^k {{ \ln }({p_i})}$, which is relatively conservative due to accumulation of *df*’s.

### Functional genetic analyses

We investigated expression patterns of 22 genes located within the *RALY*-*UQCC* region in 6 human skin melanocytic cell lines derived from donors with different skin color (lightly pigmented LP22 and LP89, moderately pigmented MP01 and darkly pigmented DP74, DP80 and DP83), in a set of 29 skin samples derived from donors with either light (*n* = 17) or dark (*n* = 12) skin pigmentation. Leftover patient skin material was collected under informed consent and with approval from the Medical Ethics Committee (METC) of Erasmus MC. Details about the cell lines, the skin samples and the methods have been described previously (Visser et al. 2014). In brief; the cell lines were grown following the manufacturer’s instructions (Cascade Biologics, Invitrogen), RNA and DNA were co-extracted using TriPure Isolation Reagent, followed by a purification step (OneStep™ PCR Inhibitor Removal Kit, Zymo Research Corporation) to remove melanin. The skin epidermal and dermal samples were obtained by separating the epidermal layer from surgically removed skin biopsies, RNA and DNA were co-extracted using Qiagen Allprep mini kit, followed by the above-described purification step to remove melanin. The reverse transcriptase (RT) reaction was performed using RevertAid™ H Minus First Strand cDNA Synthesis Kit (Fermentas GmbH) according to the manufacturer’s instructions. Quantitative real-time PCR reactions for gene expression analysis were performed using the iTaq Universal SYBR Green Supermix (Bio-Rad Laboratories). RNA sequencing was performed using a PGM (Life Technologies). RNA samples obtained from the 6 melanocyte cell lines were first treated with RiboMinus Eukaryote kit v2 (Life Technologies) to remove rRNA, after which the whole-transcriptome libraries were constructed using the Ion Total-RNA Seq Kit v2 (Life Technologies). Snapshot analysis was used to genotype the skin color-associated SNPs. Primer sequences are available on request.

We profiled the chromatin of region 20q11.22 spanning the 22 genes (*RALY*-*UQCC*) harboring the identified associated skin color SNPs. We considered several data sets that represent features associated with regulatory regions: ChIP-seq analysis in a lightly pigmented melanocytic cell line (LP22), a darkly pigmented melanocytic cell line (DP74) (Palstra et al. manuscript in preparation), and in a normal human epidermal keratinocytic cell line [NHEK (Rosenbloom et al. 2013)] of acetylated histone H3 (H3K27Ac), an active chromatin mark (Creyghton et al. 2010), DNaseI hypersensitive sites in epidermal skin melanocytes and in the NHEK cell line (Rosenbloom et al. 2013); ChIP-seq data for the transcription factor MITF in melanocytic cells (Strub et al. 2011), MITF is the melanocyte master regulator (Levy et al. 2006), ChIP-seq data in MALME-3 M melanoma cells for the transcription factor YY1 (Li et al. 2012), an ubiquitously expressed transcription factor that was reported to play an important role in melanocyte development by interacting with the melanocyte-specific isoform of MITF (Li et al. 2012); predicted melanocyte-specific enhancers (Gorkin et al. 2012) and Phastcons conserved elements inferred from 46-way alignments of placental mammals (Siepel et al. 2005).

## Electronic supplementary material

Supplementary material 1: Figure S1. Manhattan plot of GWAS results for skin color phenotypes in the Rotterdam Study, the Brisbane Twin Nevus Study, and the TwinsUK study. A, quantitative skin color saturation extracted from digital photos in the Rotterdam Study (RS, n = 5857); B, 3-level (very white, white, white-to-olive) perceived skin darkness in the Rotterdam Study (RS, n = 5857); C, 3-level (fair/light, medium, or olive/dark) perceived skin darkness in the Brisbane Twin Nevus Study (BTNS, n = 3456); D, Fitzpatrick scale of sensitivity to sun (6 levels) in the TwinsUK study (n = 2668). The –log10 p-values of all SNPs are plotted against their physical positions over the genome (hg19). The blue and red horizontal lines stand for the p-value thresholds of 1 × 10^−5^ and 5 × 10^−8^, respectively. Known pigmentation genes in the regions showing significant (p-value <5 × 10^−8^) association are highlighted in red color (TIFF 257 kb)

Supplementary material 2: Figure S2. Spatial distribution of a genetically inferred skin color score in 940 samples from 54 populations of the HGDP-CEPH. The skin color score for 940 world-wide subjects was calculated as the sum of the number of darker skin-associated alleles weighted by the regression betas for saturation using 9 SNPs from 9 gene regions (see Table 1). Yellow dots represent the geographic location of the HGDP-CEPH population samples. (TIFF 104 kb)

Supplementary material 3: Figure S3. Haplotypes associated with skin color saturation between 3184 SNPs on 20q11.2. A total of 3184 SNPs within a large region (32.3-34.0 Mb) on chromosome 20q11.2 were tested in a pair-wise manner for haplotype association with skin color saturation in the Rotterdam Study. A total of 17 SNPs with at least one P value <1 × 10^−7^ are shown. Left part: LD *r* ^2^, right part: significance of pair-wise haplotype association. (TIFF 366 kb)

Supplementary material 4: Figure S4. Chromatin profile of 22 genes on 20q11.22. IGV genome browser shows a 1.9 mb window at 20q11.22 including 22 genes (from RALY to UQCC) containing the skin-color association signals in this region. To investigate the chromatin for features of enhancer elements, the following tracks are included: ChIP-seq analysis of acetylated histone H3 (H3K27Ac), an active chromatin mark, in a lightly pigmented melanocytic cell line (LP22), a darkly pigmented melanocytic cell line (DP74) (Palstra et al., manuscript in preparation), and in a normal human epidermal keratinocytic cell line, DNaseI hypersensitive sites in epidermal skin melanocytes and in the NHEK cell line; ChIP-seq data for the transcription factor MITF in melanocytic cells, MITF is the melanocyte master regulator, ChIP-seq data in MALME-3 M melanoma cells for the transcription factor YY1, an ubiquitously expressed transcription factor that was reported to play an important role in melanocyte development by interacting with the melanocyte-specific isoform of MITF; predicted melanocyte-specific enhancers and Phastcons conserved elements inferred from 46 way alignments of placental mammals. See the method section for details about data sources. (TIFF 1282 kb)

Supplementary material 5: Supplementary Tables from Table S1 to Table S9. (XLSX 142 kb)
