# Supplementary figures and images for: Genetics of skin color variation in Europeans: genome-wide association studies with functional follow-up
Source: Hum Genet. 2015 May 12;134(8):823–35. doi: 10.1007/s00439-015-1559-0 (PMC4495261; doi:10.1007/s00439-015-1559-0)

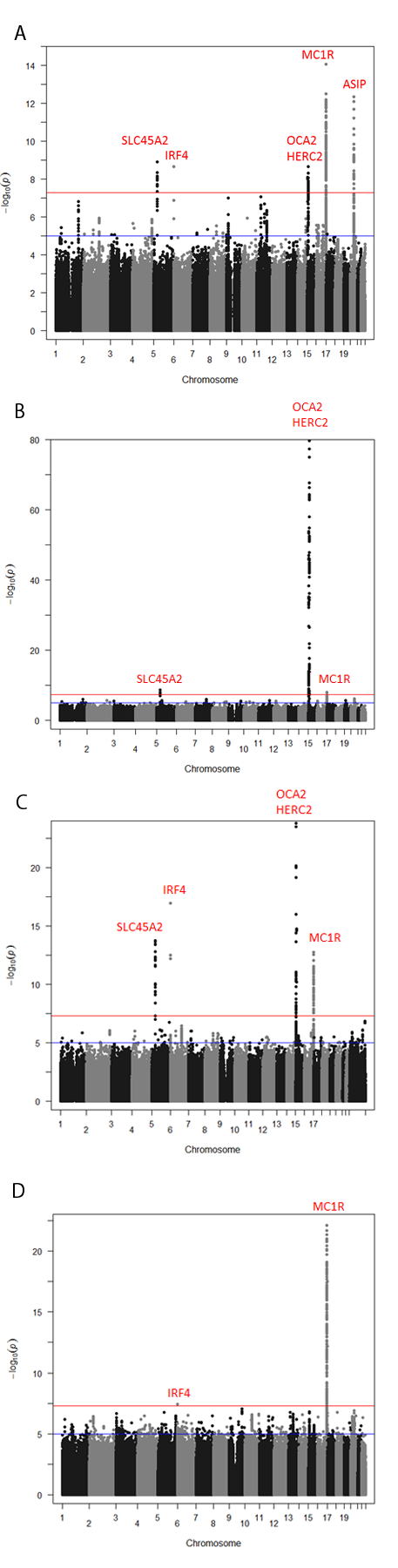

Supplement: Supplementary file 1 — Supplementary material 1: Figure S1. Manhattan plot of GWAS results for skin color phenotypes in the Rotterdam Study, the Brisbane Twin Nevus Study, and the TwinsUK study. A, quantitative skin color saturation extracted from digital photos in the Rotterdam Study (RS, n = 5857); B, 3-level (very white, white, white-to-olive) perceived skin darkness in the Rotterdam Study (RS, n = 5857); C, 3-level (fair/light, medium, or olive/dark) perceived skin darkness in the Brisbane Twin Nevus Study (BTNS, n = 3456); D, Fitzpatrick scale of sensitivity to sun (6 levels) in the TwinsUK study (n = 2668). The –log10 p-values of all SNPs are plotted against their physical positions over the genome (hg19). The blue and red horizontal lines stand for the p-value thresholds of 1 × 10^−5^ and 5 × 10^−8^, respectively. Known pigmentation genes in the regions showing significant (p-value <5 × 10^−8^) association are highlighted in red color (TIFF 257 kb) [file 439_2015_1559_MOESM1_ESM.tif]

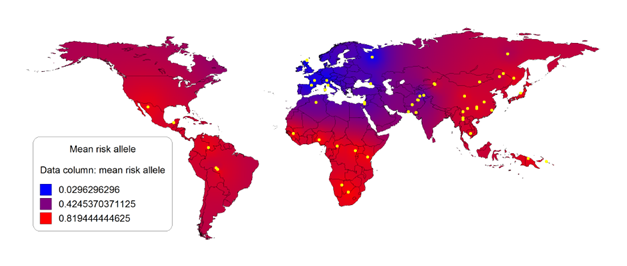

Supplement: Supplementary file 2 — Supplementary material 2: Figure S2. Spatial distribution of a genetically inferred skin color score in 940 samples from 54 populations of the HGDP-CEPH. The skin color score for 940 world-wide subjects was calculated as the sum of the number of darker skin-associated alleles weighted by the regression betas for saturation using 9 SNPs from 9 gene regions (see Table 1). Yellow dots represent the geographic location of the HGDP-CEPH population samples. (TIFF 104 kb) [file 439_2015_1559_MOESM2_ESM.tif]

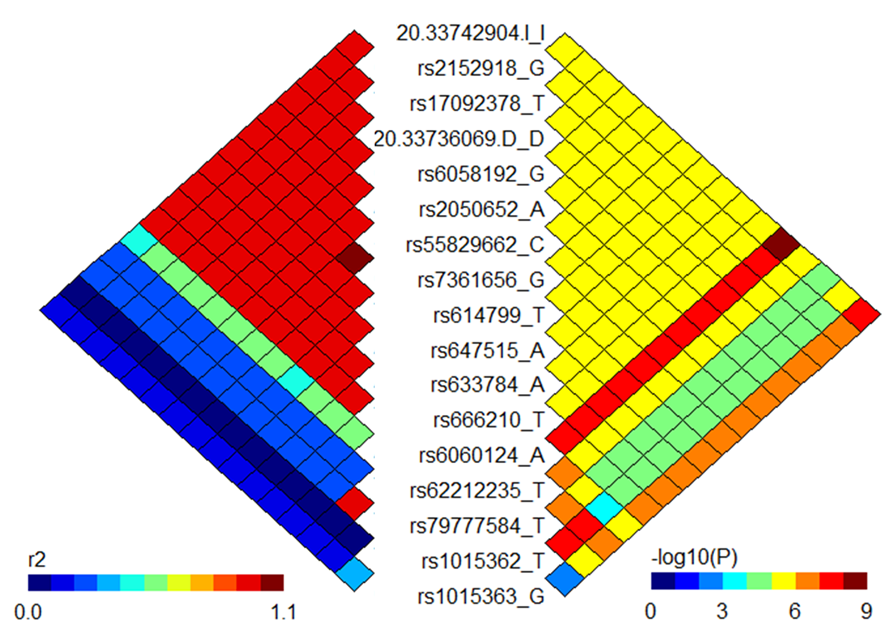

Supplement: Supplementary file 3 — Supplementary material 3: Figure S3. Haplotypes associated with skin color saturation between 3184 SNPs on 20q11.2. A total of 3184 SNPs within a large region (32.3-34.0 Mb) on chromosome 20q11.2 were tested in a pair-wise manner for haplotype association with skin color saturation in the Rotterdam Study. A total of 17 SNPs with at least one P value <1 × 10^−7^ are shown. Left part: LD r 2, right part: significance of pair-wise haplotype association. (TIFF 366 kb) [file 439_2015_1559_MOESM3_ESM.tif]

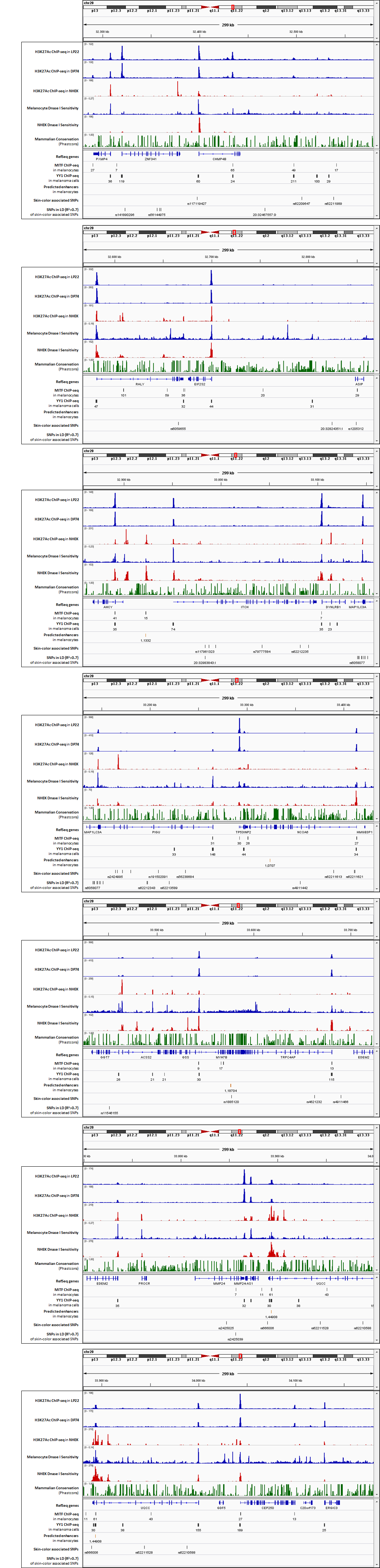

Supplement: Supplementary file 4 — Supplementary material 4: Figure S4. Chromatin profile of 22 genes on 20q11.22. IGV genome browser shows a 1.9 mb window at 20q11.22 including 22 genes (from RALY to UQCC) containing the skin-color association signals in this region. To investigate the chromatin for features of enhancer elements, the following tracks are included: ChIP-seq analysis of acetylated histone H3 (H3K27Ac), an active chromatin mark, in a lightly pigmented melanocytic cell line (LP22), a darkly pigmented melanocytic cell line (DP74) (Palstra et al., manuscript in preparation), and in a normal human epidermal keratinocytic cell line, DNaseI hypersensitive sites in epidermal skin melanocytes and in the NHEK cell line; ChIP-seq data for the transcription factor MITF in melanocytic cells, MITF is the melanocyte master regulator, ChIP-seq data in MALME-3 M melanoma cells for the transcription factor YY1, an ubiquitously expressed transcription factor that was reported to play an important role in melanocyte development by interacting with the melanocyte-specific isoform of MITF; predicted melanocyte-specific enhancers and Phastcons conserved elements inferred from 46 way alignments of placental mammals. See the method section for details about data sources. (TIFF 1282 kb) [file 439_2015_1559_MOESM4_ESM.tif]
